# Soil Giant Phage: Genome and Biological Characteristics of *Sinorhizobium* Jumbo Phage

**DOI:** 10.3390/ijms25137388

**Published:** 2024-07-05

**Authors:** Alexandra P. Kozlova, Victoria S. Muntyan, Maria E. Vladimirova, Alla S. Saksaganskaia, Marsel R. Kabilov, Maria K. Gorbunova, Andrey N. Gorshkov, Mikhail P. Grudinin, Boris V. Simarov, Marina L. Roumiantseva

**Affiliations:** 1 Laboratory of Genetics and Selection of Microorganisms, Federal State Budget Scientific Institution All-Russia Research Institute for Agricultural Microbiology (FSBSI ARRIAM), 196608 Saint Petersburg, Russia; alexsandrak95@mail.ru (A.P.K.); vucovar@yandex.ru (V.S.M.); mariiacherkasova@arriam.ru (M.E.V.); allasaksaganskaya@mail.ru (A.S.S.); gorbunovamk1@gmail.com (M.K.G.); simarov.boris@yandex.ru (B.V.S.); 2 SB RAS Genomics Core Facility, Institute of Chemical Biology and Fundamental Medicine, Siberian Branch of the Russian Academy of Sciences, 630090 Novosibirsk, Russia; kabilov@niboch.nsc.ru; 3 Smorodintsev Research Institute of Influenza, Ministry of Health of the Russian Federation, 197376 Saint Petersburg, Russia; angorsh@yahoo.com (A.N.G.); mikhail.grudinin@influenza.spb.ru (M.P.G.)

**Keywords:** jumbo rhizobiophage, *Sinorhizobium* spp., lytic activity, MOI, transmission electron microscopy, phage tRNA, cas4

## Abstract

This paper presents the first in-depth research on the biological and genomic properties of lytic rhizobiophage AP-J-162 isolated from the soils of the mountainous region of Dagestan (North Caucasus), which belongs to the centers of origin of cultivated plants, according to Vavilov N.I. The rhizobiophage host strains are nitrogen-fixing bacteria of the genus *Sinorhizobium* spp., symbionts of leguminous forage grasses. The phage particles have a myovirus virion structure. The genome of rhizobiophage AP-J-162 is double-stranded DNA of 471.5 kb in length; 711 ORFs are annotated and 41 types of tRNAs are detected. The closest phylogenetic relative of phage AP-J-162 is *Agrobacterium* phage Atu-ph07, but no rhizobiophages are known. The replicative machinery, capsid, and baseplate proteins of phage AP-J-162 are structurally similar to those of *Escherichia* phage T4, but there is no similarity between their tail protein subunits. Amino acid sequence analysis shows that 339 of the ORFs encode hypothetical or functionally relevant products, while the remaining 304 ORFs are unique. Additionally, 153 ORFs are similar to those of Atu_ph07, with one-third of the ORFs encoding different enzymes. The biological properties and genomic characteristics of phage AP-J-162 distinguish it as a unique model for exploring phage–microbe interactions with nitrogen-fixing symbiotic microorganisms.

## 1. Introduction

Bacteriophages are abundant in all the ecosystems of the Earth and their numbers can exceed those of bacteria by more than ten times, ranging from 10^3^ to 10^9^ depending on their ecological niche [1,2,3,4,5]. Phages have been found in fresh and seawater, and they have been isolated from hot water sources where temperatures can reach 76 °C. They have also been isolated from desert ecosystems, as well as from low-temperature ecosystems in the Arctic and Antarctic [6]. The composition and abundance of phage communities have been shown to correlate with variations in the soil parameters, such as the moisture, pH, organic matter availability, and bacterial community composition and density [1,4,7]. The highest diversity of viral particles has been shown in the root zone and directly in the rhizosphere of plant roots [8,9]. The latter is relevant because phages influence and/or regulate the population density of the host bacteria, which include economically valuable nitrogen-fixing microorganisms, symbionts of legume plants.

It is known that bacteriophages can have a diverse range of hosts. Phages are capable of infecting a wide range of host bacteria (polyvalent bacteriophages) that belong to different species and even to different genera [10]. However, phages are also known to infect a limited number of bacterial species of the same genus, such as *Vibrio* phage vB_VhaS-VHB1 [11], or *Agrobacterium* phage Atu_ph07, which only lyses *Agrobacterium tumefaciens* strains [12]. Furthermore, it has been shown that not all the strains of the same species can be infected, as in the case of *Sinorhizobium* phage AP-16-3 infecting *Sinorhizobium meliloti* rhizobia [13].

Bacteriophage genomes can vary in size by hundreds of times, from 2.5 to 735 kb. Phages with a genome size of more than 200 kb are described in the literature as giant, mega, or jumbo phages [14]. The isolation of phages with a large genome is associated with a number of methodological problems [15]; data on them have mainly been generated from the analysis of metaviromes from different ecosystems [16,17,18,19]. The NCBI Virus database contains about 100 studied phage genomes, the largest of which is 735 kb in size [15,16]. Fifteen giant or mega phages have now been described [16]; however, the lytic activity has only been evaluated in three phages, including *Salicola* phage SCTP-2 (440,001 bp; GenBank: MF360958.1), lysing the halophilic bacterium *Salicola* [20]; *Agrobacterium* phage Atu_ph07 (490,380 bp; NCBI RefSeq: NC_042013.1), lysing *Agrobacterium* strains [12] and *Bacillus* phage G (497,513 bp; NCBI RefSeq: NC_023719.1), effectively lysing only *Lysinibacillus* strains [21].

Due to the dramatic increase in genomic and metaviral studies, the classification of bacteriophages is undergoing significant changes and the terms “myovirus”, “siphovirus”, and “podovirus”, which were previously used to designate different classes of phages, have been proposed by the International Committee on Taxonomy of Viruses (ICTV) to be used solely to describe phage morphotypes [22,23]. Jumbo and mega phages have been shown to have a head of icosahedral symmetry and a tail of myovirus or siphovirus structure, based on which they are assigned to the class *Caudoviricetes*. Unusual variations in the tail morphology have been shown for these phages, such as the presence of massive flexible tail fibers, as in the *Sphingomonas* phage PAU, long whiskers, as in the case of the *Pectobacterium* phage CBB, or hair-like fibers arising from both the head and tail sheath, as shown for the *Escherichia* phage 121Q and *Agrobacterium* phage Atu_ph07 [12,18]. It has been suggested that these overlying structures may contribute to a stronger attachment of the phage to the host cell, which is particularly relevant for phages living in aquatic environments.

The genomes of bacteriophages have been characterized as “mosaic”, which appears to be a result of their life cycle. For example, phages can hijack single genes of the host bacterium as a result of the lysogenic cycle and thus participate in horizontal gene transfer [13]. At present, bacteriophage genomes, as well as bacterial genomes, are conceptually subdivided into two sub-parts, namely the core genes and the accessory genes. The former are essential for the complex functioning of the virus, such as the genes responsible for virion structure, viral DNA replication, and protein synthesis, and the genes whose products are required for lysis of the bacterial cell [24]. The accessory genes are not shared by related groups of phages [24,25,26]. Unlike bacteriophages with a small genome, jumbo phages contain more genes, including genes responsible for genome replication and nucleotide metabolism, and some contain multiple DNA and RNA polymerase genes. For example, the *Vibrio* phage KPV40 contains five genes that presumably encode pyridine nucleotide metabolism proteins [27]. Jumbo phages may have their own CRISPR-Cas systems, which are able to repress host transcription factors and translational processes to redirect biosynthesis to phage reproduction [16]. However, the functional and evolutionary significance of giant phage genomes remains generally unclear, as does their involvement in horizontal gene transfer.

Rhizobiophages are bacteriophages that infect nodule bacteria that are symbionts of leguminous grass and grain legume plants [28]. It is known that phages can contribute to the elimination of 4 to 50% of the total bacterial population [3], making them an economically significant factor for farmers to consider. Most of the characterized rhizobiophages belong to double-stranded DNA phages, and their genomes are 98.2 ± 58.2 kb in size. Rhizobiophages have myo-, sipho-, or podovirus morphology, varying considerably in their morphology, and belong to different families of the class *Caudoviricetes* [13,29,30]. The largest phage for bacteria of the genus *Sinorhizobium* was described as *Sinorhizobium* phage phiN3, with a genome size of 206,713 bp [31]. This phage belongs to the genus *Emdodecavirus*, but its characteristics have not been described.

In this paper, we present, for the first time, data on the jumbo rhizobiophage AP-J-162, with a genome size of 471,510 bp, which was isolated from the soils of the mountainous region of Dagestan in the northern Caucasus. We describe its biological properties, predict the structure of the core and accessory parts of the jumbo rhizobiophage genome, and provide an opinion on its potential role in horizontal gene transfer.

## 2. Results

**Figure 1:**
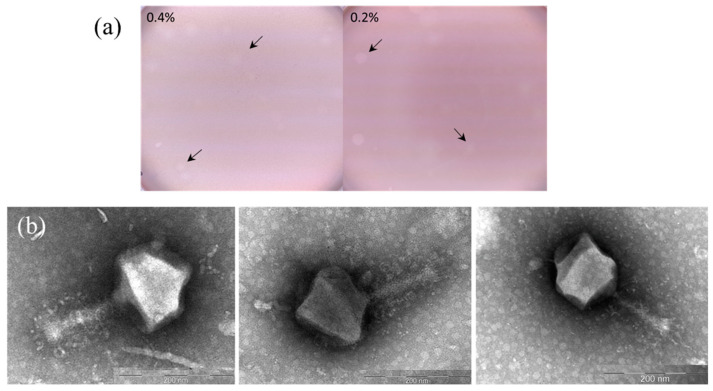
Morphological and transmission electron microscopy (TEM) characteristics of the rhizobiophage AP-J-162. (**a**) Plaques formed by the phage (indicated by arrows) on a lawn of *S. meliloti* Md3/4 on 0.2% or 0.4% semi-solid agar plates; and (**b**) microphotographs of phage AP-J-162 particles.

The soil phage AP-J-162 was isolated using a group of nine phage-sensitive test strains of *Sinorhizobium meliloti* that were grown on LA medium (see Section 4), but the ideal host was *S. meliloti* strain Md3/4. Phage AP-J-162 was found to form weak opaque plaques with a lysis zone of up to 3 mm in diameter on 0.4% agar plates and larger plaques on 0.2% semi-solid agar within 32 h (Figure 1a).

Using transmission electron microscopy (TEM) of the virions, we found that phage AP-J-162 had an icosahedral head (length 130 ± 5 nm and width 140 ± 5 nm) and a long tail (140 ± 5 nm; Figure 1b). The presence of virions with shorter tails that averaged 110 ± 5 nm in length in the TEM microphotographs suggested that the phage tail could contract (Figure 1b). According to the morphological analysis, phage AP-J-162 has a myovirus morphotype.

Phage AP-J-162’s latent period, or the time it takes for newly released phages, was 70 min and the average burst size of phage AP-J-162, or the number of released phages per cell, was estimated to be 10 PFU/mL, as determined by the one-step growth experiment (Figure S1).

The lytic activity of rhizobiophage AP-J-162 was evaluated via the classical spot test method using 48 strains of *Sinorhizobium* spp. and *Agrobacterium radiobacter*, which were grown on LA medium (see Section 4). It was found that phage AP-J-162 was able to lyse 19 out of 44 strains (43.2%) of *S. meliloti* of different geographical origins, but only 1 out of 4 *S. medicae* strains, and it did not lyse the *A. radiobacter* 204 strain. Consequently, the jumbo phage had selective activity toward *S. meliloti* strains as well as toward the closely related *S. medicae* strains (Figure S2).

**Figure 2:**
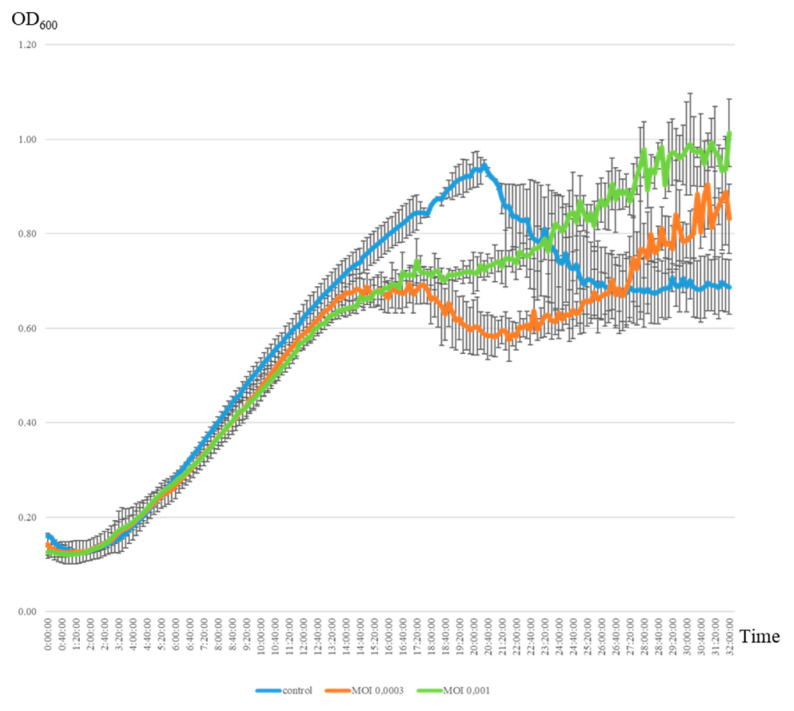
Lytic activity of jumbo phage AP-J-162 on a culture of phage-sensitive *S. meliloti* strain Md3/4 at different MOIs. The control was the growth curve of an uninfected bacterial strain (blue line), while the remaining curves represent the growth of the phage-infected strain at MOIs 0.0003 and 0.001 (orange and green lines, correspondingly).

The lytic activity of the jumbo phage AP-J-162 was evaluated toward a phage-sensitive (plaque-positive) strain of *S. meliloti* Md3/4 (see Mat. Met.). The multiplicity of infection (MOI) of phage AP-J-162 was evaluated at two different initial ratios (0.0003 and 0.001), corresponding to approximately 144,000 and 36,000 phage particles and 1.12×10^8^ bacterial cells (see Mat. Met.). Analysis of the growth curves of the uninfected control bacterial culture revealed three distinct phases of growth: the lag phase, the log phase, and the stationary phase, with durations of 3.5 h, 16 h, and 1 h, respectively, the latter of which began 20 h after the start of incubation (Figure 2, Table S1). The phage–microbial interaction evaluation of the infected and uninfected bacterial cultures showed that the start of the lag and log phases and the duration of the lag phase did not significantly differ, and these parameters did not depend on the MOI (Figure 2, Table S1). It was detected that the phage caused cell lysis within 6 h of adding the phage particles and was not dependent on the MOI (see Mat. Met; Figure 2). Phage infection led to a 1.7-fold reduction in the log phase duration and to the stationary phase starting 6 h earlier. These differences were observed at both the initial MOIs, but a larger number of phage particles (MOI of 0.001) resulted in a 2-fold prolongation of the stationary phase duration. The optical density of the infected bacterial culture in the stationary phase was similar at both initial MOIs (OD_600_ = 0.6813), but the OD_600_ values of these cultures were, on average, 27.3% (0.2545 ± 0.008) lower than in the control. Thus, the increase in the phage particle number of approximately 4-fold had no significant effect on the value of the optical density of the bacteria, but it had a significant effect on the log and stationary phase durations and the start of the stationary growth phase.

Secondary growth of the phage-infected bacterial culture was observed 20 h after infection (at both MOIs). The final optical density of the infected bacterial cultures was at a similar level 32 h after infection and averaged 0.914 in both cases. Continued growth of the bacterial culture may be associated with the generation of phage-resistant cells, which is consistent with the data obtained for phage lambda [32]. Thus, the results of the phage–microorganism interaction in liquid medium show that the density of viable bacterial cells remained quite high despite the negative selective pressure from the phage.

The estimation of the phage’s lytic activity using two methodological approaches clearly showed that the formation of plaques on solid medium did not result in the complete lysis of the bacterial cells of the phage-sensitive strain. As a result of the negative selective pressure of the phage, the bacterial cell titer decreased significantly, but the proportion of viable cells remained quite high, as evidenced by the results of the evaluation of the phage–microbial interactions in liquid culture medium.

**Figure 3:**
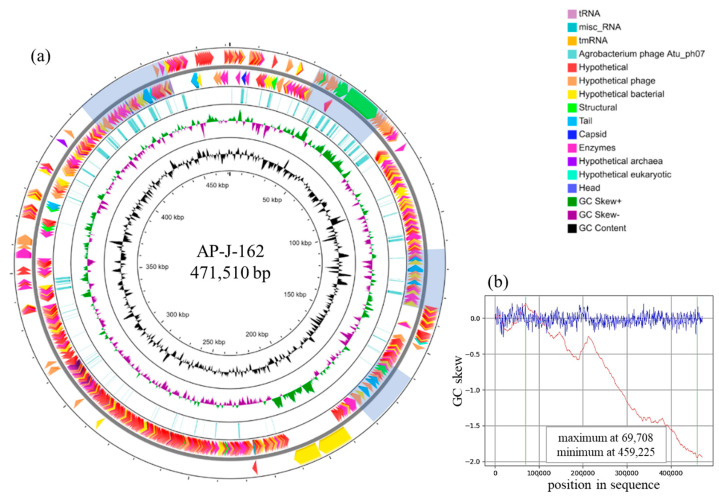
Genomic characteristics of the jumbo rhizobiophage AP-J-162. (**a**) Circular physical map of the AP-J-162 genome. The regions marked in blue represent putative clusters of structural proteins; and (**b**) GC skew diagram (blue lines) and calculated cumulative GC skew (red lines).

The genome of rhizobiophage AP-J-162 was sequenced, assembled and annotated, and its size was estimated to be 471,510 bp (Table 1). A circular physical map of the AP-J-162 genome is presented in Figure 3a. The G + C content was 47.13%, which is higher than the average values for giant phages (39.6%) according to [16,33]. The GC skew was determined, which made it possible to show that the replication pattern of phage AP-J-162 was unidirectional, which is consistent with [16] (Figure 3b).

A total of 711 ORFs were identified in the phage AP-J-162 genome, of which no similar nucleotide sequences were identified for 666 ORFs in the NCBI database. Nucleotide sequence similarities were shown for 22 ORFs that encoded tRNAs and for 23 ORFs that were predominantly similar to those of bacteria (13 of the 23 ORFs), as well as to the sequences of phages and single representatives of eukaryotes from different kingdoms (4 and 6 ORFs, respectively), via BLASTN (identity: 72.08–100%, cover: 1–99%).

An analysis of 22 nucleotide sequences encoding 19 different types of tRNAs showed that the ORFs were predominantly similar to sequences encoding tRNAs typical of bacteria (14 of 22 ORFs; identity: 81.82–100%, cover: 92–100%). Only in three cases were the sequences similar to those of the tRNAs of other phages (identity: 89.47–96.05%, cover: 82–100%), and in single cases, there were ORFs similar to those encoding tRNAs of representatives of eukaryotes of different kingdoms (identity: 94.59–100%, cover: 36–48%). In the BLASTN analysis, only 23 ORFs with similar sequences and not encoding tRNAs were identified. Among them, only two sequences had a coverage of more than 50%: thymidylate synthase from the *Agrobacterium tumefaciens* strain Kin002 (identity: 72.08%, cover: 99%) and capsid vertex protein from an uncultured *Caudovirales* phage (identity: 72.97%, cover: 62%).

Annotation of the AP-J-162 phage genome revealed another 44 sequences that encode tRNAs belonging to 22 different types. In addition, sequences encoding tmRNA (SsrA—transfer–messenger RNA) and misc. RNA were identified (Table 1).

As a result, the phage AP-J-162 genome contains 66 ORFs that encode 41 types of tRNAs, according to tRNA Scan, which were represented by one or two copies (frequencies of 0.51 and 0.39, respectively), and in single cases, by three and four copies (0.07 and 0.02, respectively). These 41 types of tRNAs corresponded to codons of 19 of the 20 canonical amino acids, but one was a non-canonical amino acid. No ORF encoding tRNA^Tyr^ was identified, but a sequence encoding tRNA^Pyl^ (pyrrolysine) was identified in which the CUA anticodon corresponded to the UAG stop codon, which is common in methanogenic archaea (Table S2; [34]).

The codon usage frequencies were estimated for the 643 protein-coding ORFs of phage AP-J-162, as well as for the core genes of the bacteria hosts, according to [35,36]. No tRNAs were identified for 15 amino acid codons in either the phage AP-J-162 or the *S. meliloti* genome (Table S2). This fact does not contradict the data of other authors, since the codons for which tRNAs are absent can be recognized by other tRNAs in accordance with the Wobble hypothesis [37,38,39].

It was found that 35 tRNAs out of the 41 mentioned above were present in both phage AP-J-162 and *S. meliloti*. The frequency of codon usage corresponding to 21 of the 35 tRNAs was higher in the case of bacteria, whereas in the case of the phage, the frequency of codon usage was higher for the other 14 tRNAs. For example, the frequency of codons’ usage of the ACA was 7.5 times higher in the phage than in the bacteria (0.3 and 0.04, respectively), and conversely, the frequency of codons’ usage in the case of CCG was 3.5 times less frequent in the phage than in the host bacterium (0.20 and 0.69, respectively), and the corresponding tRNAs were tRNA^Thr^ (UGU) and tRNA^Pro^ (CGG) (Table S2).

Six tRNAs (tRNA^Phe^ (AAA), tRNA^Ile^ (UAU), tRNA^Arg^ (UCG), tRNA^Ser^ (ACU), tRNA^Thr^ (CUC), and tRNA^Pyl^ (CUA)), which were present in only the phage AP-J-162 genome (Table S2), were identified. It should be pointed out that the differences between phage AP-J-162 and *S. meliloti* in the occurrence of codons for each of the corresponding amino acids were significant (*p* < 1 × 10^−4^).

Thus, the phage AP-J-162 genome contains numerous tRNAs, including phage-specific tRNAs, and the determined variation in codon usage between the genes of the phage and the host bacterium strongly indicates that such structural and functional specificity of the phage genome is the result of phage–microbial evolutionary mutual interaction. This determines the most efficient optimization of mRNA decoding and phage biosynthesis, which is in agreement with the data of other authors [40,41].

An analysis of the amino acid sequences of the 643 protein-coding phage AP-J-162 ORFs revealed that 304 ORFs were unique, and no similar sequences were identified in the NCBI database. The products of another 191 ORFs had similarities to the hypothetical proteins, and these ORFs had phage and bacterial origins (100 and 86 ORFs, respectively); individual ORFs were similar to the products of archaea and fungi (4 and 1 ORF, respectively). The functional significance of their products was predicted for only 148 ORFs (Table 1). The products of 152 of the 339 ORFs (44.8%) were identified as hypothetical or were predicted to be similar to those of *Agrobacterium* phage Atu_ph07. These ORFs were composed of 21 ORFs (13.8%) encoding for viral particle structural proteins, 54 ORFs (35.5%) encoding various enzymes (see below), and the products of the remaining ORFs (50.7%) were hypothetical proteins.

Of the 148 ORFs above, 30 encoded structural elements and products affecting phage particle morphogenesis (Table 2). Moreover, 21 of the 30 ORFs identified were similar to those in *Agrobacterium* phage Atu_ph07, and almost half of them encoded tail fibers, tail envelope proteins, and unidentified tail proteins. Three of the five tail fiber proteins (gp145, 221, 569) of phage AP-J-162 had the highest similarity to those of phage Atu_ph07 (identity: 33%, cover: up to 98%, e-value: 2 × 10^−24^). This fact may be the basis for the assumption that phage AP-J-162 can infect *Agrobacterium* cells, which, however, was not confirmed when the lytic activity of the phage was assessed (see above).

The other 9 out of the 30 ORFs were similar to the ORFs of myovirus phages and phiCbK siphovirus, and to the ORFs of various bacteria, including representatives of *Ectothiorhodospiraceae*, a family of purple sulfur bacteria, the deep-sea bacterium *Flammeovirga* sp., the betaproteobacteria *Thiobacillus thioparus* and *Pseudomonas* sp., and the unique ORFs similar to ORFs of the fungus *Verticillium longisporum*, a plant pathogen. The products of these ORFs were tail fiber proteins, major capsid proteins (gp9), outer membrane proteins (gp184), and potential structural proteins (Table 2). The closer structural analogs were identified for a number of ORF products of phage AP-J-162 (Table 2). Thus, the structure of hemagglutinin (gp213) was similar to that of human integrin, while the structure of the portal protein (gp29) of the major capsid protein (gp009) and baseplate subunits were similar to those of phage T4. The tail fiber proteins had a structural similarity to the *Drosophila melanogaster* apoptosome, as well as to *Sus scrofa* adenosine triphosphatase and to *Clostridioides difficile* toxin B (gp145, gp221, and gp569) (Table 2).

The group of ORFs encoding structural elements also included 32 ORFs from the group encoding hypothetical products (Table 1) and 3 ORFs from the group with predicted function, the products of which are hypothetical enzymes. However, in the case of phage Atu_ph07, all 35 of the above ORFs were predicted to be structural based on electrospray ionization mass spectrometry (ESI-MS/MS) data, according to [12].

Thus, the ORFs that preferentially encode structural proteins of the AP-J-162 phage virion were grouped into four clusters, namely ORFs 49–63, ORFs 137–156, ORFs 213–223, and ORFs 647–675 (Figure 3a).

A total of 118 of the 148 ORFs were predicted to encode enzymes, and of these, 54 ORFs were similar for both phages (Table 1). All 118 ORFs were assigned to 21 COG categories, of which O, L, and J were the most abundant (Table S3).

The COG category L (replication, recombination, and repair) contained 23 ORFs, of which 14 ORFs were similar to those of phage Atu_ph07 (identity: 39.7–64.55%, cover: 93–100%), 7 ORFs were similar to those of six different species of bacteria (identity: 29.23–62.5%, cover: 85–100%), 1 ORF (gp645) was similar to that of uncultured *Caudovirales* phage (identity: 70.43%, cover: up to 92%), and a unique ORF had a product that was presumed to have catalytic activity (Table S2). The ORFs of phage AP-J-162, similar to those of phage Atu_ph07, encoded DNA polymerase III subunits (gp197, 206) (identity: up to 50.1%, cover: up to 100%), as well as ORFs encoding WXY group proteins, including UvsW helicase (gp638), recombination, repair, and ssDNA binding protein UvsY (gp643 and gp645), and the above ORF encoding RecA/RadA recombinase. All of the above three ORFs are reported to be responsible for DNA repair in bacteriophage T4 [42]. The products of the three other ORFs, one of which (gp599) is related to phage Atu_ph07 and the other two (gp035 and gp054) are similar to those of bacteria, are believed to have exonuclease activity (cover: up to 97%, identity: up to 62.5%; Table S3). An ORF (gp127, identity: 52%, cover: 100%) was identified and found to be similar to that encoding a hypothetical nucleotide-binding protein of *Verrucomicrobiaceae* bacterium, which is a Gram-negative anaerobic member of the human gut microflora capable of degrading mucin [43] (Table S3). Based on the data we obtained (GC skew, ORF composition), we suggested that for the phage AP-J-162 genome, the process of unidirectional replication is most likely. Unidirectional replication is realized by either the “rolling circle” mechanism, characteristic of small phage and plasmid genomes, or by mechanism recombination-dependent DNA replication (RDR), according to [44]. According to our prediction, the phage AP-J-162 genome did not contain an *ori* sequence specific to the rolling circle replication mechanism. It was found that nine ORFs encoding two subunits of DNA polymerase III (gp197, gp206), two helicases (gp288, gp638), two ligases (gp097, gp135) and three terminases (gp002, gp005, gp648) were annotated in the phage AP-J-162 genome. The RDR mechanism is most likely for the genome of phage AP-J-162, since it contains their own polymerases (gp112, gp127), helicases (gp288, gp638), and recombinase (gp645), as well as the recombination mediator protein UvsY (gp643).

A total of 21 ORFs belonged to the COG category O (post-translational modification, protein turnover, and chaperones), of which 9 ORFs were similar to those of phage Atu_ph07 (identity: 39.07–81.48%, cover: 88–99%), 2 ORFs were similar to those of rhizobiophages phiN3 and RHph_TM30 (identity: 56.06–64.62%, cover: 97–99%), and 9 ORFs were similar to those of seven different bacterial species (identity: 35.12–67.57%, cover: 76–98%). An ORF (gp204) similar to chaperone GroES of *Euryarchaeota* archaeon (identity: 45.4%, cover: 86%) was identified, which was also similar to a hypothetical phage Atu_ph07 protein (identity: 58.59%, cover: 88%). There were six phage ORFs encoding potential caseinolytic proteins (Clp proteins) that had been involved in protein degradation, wherein two out of six ORFs were similar to those of phage Atu_ph07, whereas the remaining four ORFs were similar to those of bacteria (Table S3). The *clpX* gene was found to be located directly adjacent to the genes encoding the translation initiation factor IF-3 (gp547) and topoisomerase (gp551, gp553), and these three ORFs belong to the COG category J. This suggests that the ClpX phage AP-J-162 protein is involved in the regulation of phage DNA replication, as was shown in the case of the ClpX/ClpP of lambda phage [45]. The ORFs encoding heat shock proteins Hsp20 (gp517) and DnaJ (Hsp40, gp155), which are involved in replication processes and defense against stress, also belonged to the COG category O. An ORF encoding protein Cas4, which is a RecB-like nuclease with a C-terminal cluster of three cysteines, was identified and is involved in the process of spacer acquisition together with Cas1 and Cas2 in CRISPR-Cas systems of types IA, IB, IC, ID, IU, and II-B. The sequence of the RecB-like nuclease with a C-terminal cluster of three cysteines (Cas4) was similar in phage AP-J-162 to that of phage Atu_ph07.

The COG category J (translation, ribosomal structure, and biogenesis) included 13 ORFs, of which 7 ORFs were similar to those of phage Atu_ph07 (identity: 37.5–68.18%, cover: 39–100%), another 4 ORFs were related to those of four species of bacteria (identity: 29.09–53.99%, cover: 61–97%), and several single ORFs were similar to those of *Erwinia* phage AH04 (gp099, identity: 46.9%, cover: 92%) and uncultured *Caudovirales* phage (gp154, identity: 47.74%, cover: 91%), which encoded N-acetyltransferase and formylmethionyl-tRNA deformylase, respectively. The ORF gp532 of phage AP-J-162, similar to that of phage Atu_ph07, encoded the 30S ribosomal protein S21, which, together with the 50S ribosomal protein L7/L12 (gp556) and translation initiation factor IF-3 (gp547), is involved in intercepting and redirecting the translation of host bacterial genes, such as competitive ribosome binding or preferential initiation of phage mRNAs, according to [16]. Among the ORFs encoding hydrolases and ligases were also ORFs (gp295, gp094) similar to those of the bacteria *Beijerinckia* sp. L45 and *Thermopetrobacter* sp. TC1, respectively. We also identified phage ORFs related to phage Atu_ph07 that encoded tRNA-processing enzymes, including the tRNA nucleotidyltransferase involved in tRNA maturation, the peptidyl-tRNA hydrolases that reduce the pool of peptidyl-tRNAs whose accumulation contributes to protein biosynthesis shutdown, and the RNA ligases involved in translation.

We searched for the lytic module of phage AP-J-162. According to the literature [46,47,48], the lytic proteins of phages include various lysines, peptidases, glycosidases, amidases, lyases, and tail protein complexes. However, among the ORFs of phage AP-J-162 encoding components of the putative lytic module, only peptidase (gp289), which removes the C-terminal amino acid residues of polysaccharides, and choline (gp138) and endolysin (gp608), which are responsible for the release of mature viral particles from the bacterial cell, were identified. Analysis of the amino acid sequence of endolysin showed that the endolysin of phage AP-J-162 presumably lyses cells via a canonical lytic mechanism in a manner similar to that of the endolysin T4L of phage T4.

Thus, functional relevance analysis and phylogenetic analysis of the ORFs showed that the core part of the AP-J-162 phage genome can include ORFs encoding structural elements of the viral particle and 54 ORFs encoding enzymes, which are similar between phages AP-J-162 and Atu_ph07. ORFs of category L, which are not similar to Atu_ph07 but are important for phage genome replication, should also be included in the core part.

A phylogenetic analysis of phage AP-J-162 was performed, considering 25 full-genome sequences of different bacteriophages from the NCBI database (accessed on 12 December 2023), whose genomes ranged from 168 to 497 kb and whose GC compositions ranged from 30 to 62%. Among the studied phages were phages lysing bacteria of the genus *S. meliloti*, phages from the genus *Emdodecavirus*, Rak2-like viruses, named after *Enterobacteria* phage Rak2 [12], the model *Escherichia* phage T4, and jumbo phages of *Pseudomonas* and *Bacillus* phage G (Table S4).

**Figure 4:**
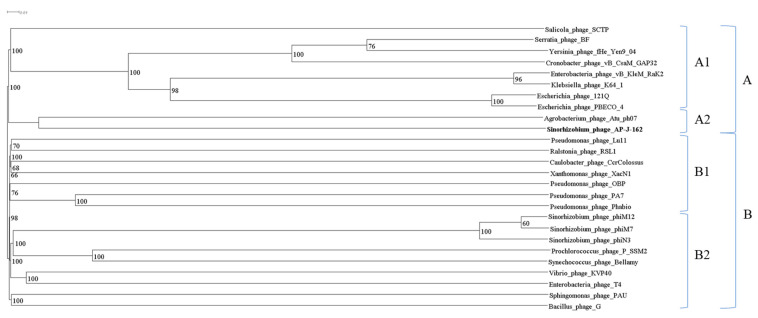
Phylogenetic tree of large-genome phages. The scale bar is 0.01 for the nucleotide substitutions per site. A and B clades; A1, A2, B1 and B2 clusters. The phylogenetic tree was constructed by the N-J method according to the criterion of balanced minimal evolution implemented by the FASTME algorithm and its improvement by topological SPR moves obtained by the D6 formula (average bootstrap 91%).

Top-down clustering revealed clusters A1 and A2 (bootstrap 100%) in clade A, which contained Rak-like phage sequences and sequences of AP-J-162 and *Agrobacterium* phage Atu_ph07, respectively. Clade B contained two clusters, B1 and B2 (bootstrap 98%). Cluster B1 united the sequences of *Pseudomonas*, *Ralstonia*, and *Caulobacter* phages, and cluster B2 combined the sequences of three rhizobiophages of the genus *Emdodecavirus* (*Sinorhizobium* phage phiN3, *Sinorhizobium* phage phiM12, and *Sinorhizobium* phage phiM7), the model phage T4, and the largest known jumbo phage *Bacillus* phage G (Figure 4, Table S4). Thus, the closest phylogenetic relative of rhizobiophage AP-J-162 is *Agrobacterium* phage Atu_ph07.

## 3. Discussion

According to metavirome studies, phages with huge genomes are widely distributed in all the ecosystems of the Earth, but their isolation involves a number of methodological difficulties. Currently, three jumbo phages with genome sizes exceeding 400 kb are known, for which data on their lytic activity are available. These are *Salicola* phage SCTP-2, which was able to infect two hosts, *Agrobacterium* phage Atu_ph07, which lysed agrobacteria, and *Bacillus* phage G, which effectively lysed only the *Lysinibacillus* strain.

For the first time, we obtained a culture of lytic jumbo rhizobiophage AP-J-162 and studied its structural, biological, and genomic characteristics. The phage was isolated from the soils of the mountainous region of Dagestan, Caucasus, the territory of which belongs to the centers of origin of cultivated plants, according to the studies of Vavilov N.I. The genome of phage AP-J-162 is represented by a circular double-stranded DNA with a length of 471.5 kb, which, according to our prediction, unidirectionally replicates in the bacterial genome (RDR mechanism). This is consistent with the data obtained for phages with large genomes [16]. The genome size of phage AP-J-162 is comparable to the size of non-symbiotic plasmids of root nodule bacteria. For example, native strains of *S. meliloti* have plasmids with sizes exceeding 400 kb.

The host strains of this rhizobiophage AP-J-162 are nitrogen-fixing symbionts of economically valuable species of legume forage grasses belonging to the genus *Sinorhizobium* spp. The phage was found to have selective lytic activity against strains of two closely related species, *Sinorhizobium meliloti* and *S. medicae*.

According to the phylogenetic analysis of the complete genome sequences of 26 giant phages whose genome size range from 168 to 497 kb, the closest phylogenetic relative of phage AP-J-162 is phage Atu-ph07, but according to the branching analysis, the mentioned phages diverged from the last common ancestral sequence quite a long time ago. The genomes of both phages are phylogenetically distant from rhizobiophages, which complete genome sequences clustered together in a distant clade. It should be noted that the host bacteria of both phages *S. meliloti* and *A. tumefaciens*, according to the literature, have a high level of similarity and belong to the same family *Rhizobiaceae* [49], but agrobacteria are not hosts for rhizobiophage AP-J-162, according to the experimental data, which is consistent with the data of the phylogenetic analysis.

The annotation revealed ORFs of phage and bacterial origin in the genome of phage AP-J-162, which occurred with similar frequencies (0.26 and 0.2, respectively). In addition, single ORFs similar to those of archaea and eukaryotes were identified. Genome annotation showed that similar sequences were identified in the database for only 45 nucleotide sequences, whereas 93.7% of phage ORFs were unique. Amino acid sequence analysis revealed that 339 of the 711 ORFs encoded hypothetical or functionally relevant products. More than 45% of these ORFs were similar to those of *Agrobacterium* phage Atu_ph07, with one-third of these ORFs encoding different enzymes. Among the ORFs of bacterial origin, ORFs encoding exonucleases, hydrolases, and ligases were identified. Sequences encoding chaperone GroES, as well as ORFs encoding potential caseinolytic proteins (Clp proteins), which have been involved in protein degradation, were also identified.

ORFs encoding structural elements of the virion of phage AP-J-162 have been analyzed to show that the capsid and baseplate of the phage are structurally similar to those of the model *Escherichia* phage T4, but no tail protein subunits common to these phages have been identified. Four clusters were identified in which ORFs encoding virion structural elements were clustered. An analysis of the replicative apparatus of phage AP-J-162 allowed us to show the presence of ORFs similar to those of *Escherichia* phage T4, which encodes DNA polymerase III subunits and encodes WXY group proteins responsible for DNA repair [42].

The codon usage frequency analysis revealed that in the AP-J-162 phage genome, a similar number but different types of tRNAs are efficiently used by the phage transcriptional apparatus and the host bacterium (20 and 21 tRNAs, respectively). Six tRNAs specific to only the phage genome were identified, as well as RNA ligases and peptidyl-tRNA hydrolases, which reduce the pool of peptidyl-tRNAs whose accumulation contributes to protein biosynthesis shutdown.

We did not obtain data that would indicate the presence of a lysogenic path for phage AP-J-162 since no proteins responsible for the integration and anchoring of phage genes in the genomes of symbiotically active bacteria *Sinorhizobium* spp. were identified. There is no sequence encoding integrase in the phage genome, and analysis of the full-genome sequences of *S. meliloti* strains available for a study similar to the study we performed earlier [13] did not reveal similar sequences in rhizobia genomes (data not presented).

The data that have been acquired to the present day demonstrate that mega phages carrying large pools of diverse genes of various phylogenetic origins represent a little-known area of both virology and microbiology. The range of hosts for known jumbo phages, according to the literature data, is rather narrow, which, firstly, indicates that there are limiting factors for maintaining their viability. At the same time, the level of autonomy of jumbo phage genomes has led to a significant decrease in their dependence on the host bacterial genome. According to recent data, the vast majority of phages can be phage–plasmids [50], which is possible and occurs in the case of jumbo phages. Therefore, on the basis of our studies, it is suggested that the set of genes in giant phages, in particular in the jumbo rhizobiophage AP-J-162, may determine their survival strategy, resulting in the high efficiency of the lytic cycle or the ability to exist in the stage of pseudolysogeny, which is consistent with the data obtained for the metagenomic studies discussed above. All this together allows us to consider phage AP-J-162 a unique model for studying evolutionary interactions with nitrogen-fixing symbiotic microorganisms under conditions of global changes in soil–climatic features.

## 4. Materials and Methods

### 4.1. Phage Isolation and Purification

*Sinorhizobium* phage AP-J-162 was isolated from soil sample (5 g) collected during the summer from the mountainous region of the North Caucasus (Dagestan, Russia, 42°19′59″ N, 47°08′59″ E), according to our previously described enrichment protocol [13], using several phage-sensitive strains according to [51].

### 4.2. Bacterial Strains and Grows Conditions

Native *S. meliloti* and *S. medicae* strains (44 and 4, correspondingly) were randomly selected from the collection of the Laboratory of Genetics and Selection of Microorganisms of FSBSI ARRIAM, which had been isolated from geographically distant regions. These rhizobial strains and *Agrobacterium radiobacter* (*A. tumefaciens*) strain 204 were used to test the bacteriophage lytic activity. The strains were cultured on LA (2%) and broth LB media at 28 °C, as well as on semi-solid (0.2–0.4%) top-layer agar for phage titration by the Adams double-layer agar method [52]. The bacterial cell density (OD_600_) was measured by a Smart Spec Plus scanning spectrophotometer (BioRad, Hercules, CA, USA).

### 4.3. Evaluation of Phage Lytic Activity (Host Range)

The lytic activity spectrum of phage AP-J-162 was assessed by the spot test method using the protocol described in [53,54] on the collection of bacterial strains described above. The dynamic of phage–microbial interactions was evaluated by the changes in the bacterial growth curves and multiplicity of phage infection (MOI). The MOI is the ratio of the number of virions (phage particles) that are added per each bacterial cells in the initial time period [55,56]. To enumerate the number of phage particles, the Adams double-layer agar method was used [52]. The estimation of the approximate number of bacterial cells was carried out using the “*E. coli* Cell Culture Concentration from OD_600_ Calculator” (https://www.agilent.com/store/biocalculators/calcODBacterial.jsp?_requestid=1240383 (accessed on 10 March 2024)), as the cell sizes of *E. coli* and *S. meliloti* are similar [57]. An overnight culture (OD_600_ = 0.1413) of the plaque-positive host strain Md3/4 [53] was used for evaluation of the phage–microbial interaction. The experiment was carried out in quadruplicate in LB broth during 32 h with a purified AP-J-162 phage lysate on a SpectraMax 190 microplate reader (Molecular Devices, San Jose, CA, USA). Phage lysate was used at two initial MOIs, 0.001 and 0.0003.

### 4.4. Transmission Electron Microscopy

For the electron microscopic examination of the phage particles, the negative contrast method was used. For this purpose, the suspension under study was adsorbed onto copper grids for electron microscopy (300 mesh) (SigmaAldrich, St. Louis, MO, USA), coated with a collodion supporting film. After adsorption of particles from the suspension onto the supporting film for 1–2 min, the grids were washed twice with distilled water. Next, the sample was negatively contrasted for 1–2 min in a 2% solution of sodium salt of phosphotungstic acid (SigmaAldrich, St. Louis, MO, USA), pH 7.2. After this, the grids were dried and examined in a JEM 1011 transmission electron microscope (JEOL, Tokyo, Japan). Electron microphotographs were obtained using a high-resolution digital camera Morada (Olympus, Tokyo, Japan) in the instrumental magnification range of 60,000×–250,000×.

### 4.5. One-Step Growth Curve of Phage AP-J-162

Phage AP-J-162’s latent period and burst size were estimated according to the protocol described in [58], with some modifications: the *S. meliloti* strains were grown at 28 °C and the total run time of the experiment was 100 min.

### 4.6. DNA Isolation, Sequencing, and Annotation of the Bacteriophage Genome

Genomic DNA of phage AP-J-162 was isolated using the GeneJET Viral DNA/RNA Purification Kit (Thermo Fisher Scientific, Waltham, MA, USA). The genomic DNA was fragmented to an average size of 600 bp using the Covaris S2 instrument (Covaris, Woburn, MA, USA).

A paired-end library was constructed using NEBNext dual-index oligonucleotide adapters and the NEBNext Ultra II DNA Library Preparation Kit for Illumina (New England BioLabs (NEB), Ipswich, MA, USA). The DNA library was sequenced using the v3 reagent kit (2 × 300 bp) on a MiSeq desktop sequencer (Illumina, San Diego, CA, USA) at the Genomics Center for Collective Use, Siberian Branch of the Russian Academy of Sciences, Novosibirsk (ICBFM Siberian Branch of the Russian Academy of Sciences), with a yield of about 1.4 million reads with paired ends. Short sequences were filtered for quality and adapter sequences were removed using the BBDuk software tool from the BBMap package (https://sourceforge.net/projects/bbmap/ (accessed on 10 October 2022)) (ktrim = r k = 23 mink = 11 hdist = 1 tpe tbo minlen = 25 qtrim = rl trimq = 10) with the default parameters. Annotation of the bacteriophage genome was performed using eggNOG-mapper v2 [59].

### 4.7. Phylogenetic Analysis

The entire analysis was carried out by the VICTOR web service (https://victor.dsmz.de (accessed on 10 December 2023)), a method for the genome-based phylogeny and classification of prokaryotic viruses [60]. All the pairwise comparisons of the nucleotide sequences were conducted using the Genome-BLAST Distance Phylogeny (GBDP) method [61] under settings recommended for prokaryotic viruses [60].

The resulting intergenomic distances were used to infer a balanced minimum evolution tree with branch support via FASTME, including SPR postprocessing [62] for the formula D6. Branch support was inferred from 100 pseudo-bootstrap replicates each. The trees were rooted at the midpoint [63] and visualized was performed with Dendroscope3 [64].

### 4.8. Analysis of Nucleotide and Amino Acid Sequences

BLAST family programs were used to identify and analyze the nucleotide and amino acid sequences, respectively, based on their similarity to sequences from the NCBI database. A physical map of the *Sinorhizobium* phage AP-J-162 genome was visualized using the Proksee server (https://proksee.ca/ (accessed on 15 March 2024)). Prediction of the three-dimensional structure models of the proteins was performed using the I-TASSER web server [65].

The search for tRNA genes in the sequences was performed using tRNAscan-SE [26]. The codon counts were determined using Sequence Manipulation Suite: Codon Usage Calculator [66]. The codon usage frequencies were determined as the proportion of the use of a specific codon of a certain amino acid from all the codons of this amino acid.

### 4.9. Nucleotide Accession

The genome sequence of AP-J-162 was deposited in the GenBank with the nucleotide accession number.

## Figures and Tables

**Table 1:** Genomic characteristics of *Sinorhizobium* phage AP-J-162 and *Agrobacterium* phage Atu_ph07.

Genome Annotation	*Sinorhizobium* Phage AP-J-162	*Agrobacterium* Phage Atu_ph07
Genome size, kb	471.5	490.4
G + C, %	47.13	37.1
ORFs:	711	714
Unique	304	390
Hypothetical proteins	191	214
Predicted function	148	110
tRNA	66	33
tmRNA	1	0
Misc. R.N.A.	1	0
ORF similar	152	
ORFs encoding:	21	
(i) structural elements of phage particle		
(ii) enzymes	54	
(iii) hypothetical proteins	77	

**Table 2:** ORFs of phage AP-J-162 encoding structural elements.

№ ORF (gp)	Length, a.a.	Predicted Function	Best Match (Identity/Cover, %)	The Closer Structural Analog Predicted with I-TASSER Web Server (AlphaFold2 Algorithm) (PDB Accession)	
9	408	Major capsid protein	*Myoviridae* sp. (77.5/97)	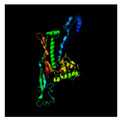	Capsid protein bacteriophage T4 (5VF3)
29	574	Portal protein	*Agrobacterium* phage Atu_ph07 (57.58/100)	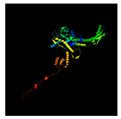	Portal protein bacteriophage T4 (3JA7)
31	279	Baseplate	*Agrobacterium* phage Atu_ph07 (50.18/98)	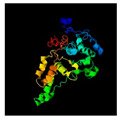	Transporter protein *Chlamydomonas reinhardtii* (5ZME)
50	103	Baseplate wedge protein	*Agrobacterium* phage Atu_ph07 (62.14/100)		
56	131	Baseplate wedge protein	*Agrobacterium* phage Atu_ph07 (51.56/97)	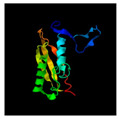	Hexagonal pre-attachment T4 baseplate–tail tube complex (5IV5)
57	1198	Baseplate wedge protein	*Agrobacterium* phage Atu_ph07 (62.35/100)		
58	4455	Structural protein	*Agrobacterium* phage Atu_ph07 (49.61/99)		
139	166	Tail protein	*Caulobacter* phage CcrColossus (33.77/87)		
141	959	Tail fiber protein	*Myoviridae* sp. (28.88/59)	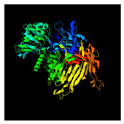	Tc toxin *Photorhabdus luminescens* (6SUF)
145	602	Tail fiber protein	*Agrobacterium* phage Atu_ph07 (31.34/98)	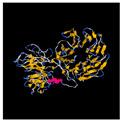	Apoptosome *Drosophila melanogaster* (4V4L)
184	248	Outer membrane protein	*Verticillium longisporum* (65.71/42)		
213	375	Hemagglutinin	*Agrobacterium* phage Atu_ph07 (31.77/99)	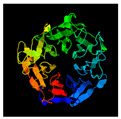	Human integrin (3VI3)
214	394	Hemagglutinin	*Agrobacterium* phage Atu_ph07 (34.91/94)		
218	311	Tail protein	*Agrobacterium* phage Atu_ph07 (45.16/99)	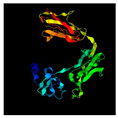	IFN-alpha/beta-binding protein C12R *Ectromelia* virus Moscow (3OQ3)
219	726	Tail protein	*Agrobacterium* phage Atu_ph07 (40.06/99)	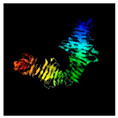	S-layer protein RsaA *Caulobacter crescentus* (8BQE)
220	679	Tail protein	*Agrobacterium* phage Atu_ph07 (34.2/98)		
221	788	Tail fiber protein	*Agrobacterium* phage Atu_ph07 (30.41/59)	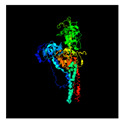	ATPase *Sus scrofa* (4HQJ)
282	171	Outer membrane protein	*Agrobacterium* phage Atu_ph07 (35.09/100)		
569	1280	Tail fiber protein	*Agrobacterium* phage Atu_ph07 (32.52/42)	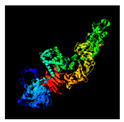	Toxin B *Clostridioides difficile* (2BVL)
572	564	Potential structural protein	*Thiobacillus* sp. (44.07/93)		
573	210	Potential structural protein	*Ectothiorhodospiraceae* bacterium 2226 (40.0/93)		
589	411	Tail protein	*Pseudomonas* sp. Fl4BN1 (38.83/69)	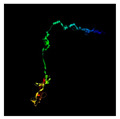	Subunit of pre-attachment T4 baseplate–tail tube complex (5IV5)
590	303	Tail protein	*Agrobacterium* phage Atu_ph07 (36.31/99)		
647	957	Potential structural protein	*Myoviridae* sp. (41.8/39)		
657	143	Head completion protein	*Agrobacterium* phage Atu_ph07 (61.76/95)	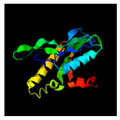	TnsA/TnsC transposition complex *Escherichia coli* (1T0F)
658	331	Potential structural protein	*Agrobacterium* phage Atu_ph07 (46.71/98)		
659	194	Potential structural protein	*Agrobacterium* phage Atu_ph07 (69.63/98)		
665	1092	Tail sheath monomer	*Agrobacterium* phage Atu_ph07 (59.85/99)	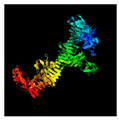	Secreted extracellular protein A (SepA) *Shigella flexneri* (5J44)
696	967	Tail fiber protein	*Flammeovirga* sp. EKP202 (42.61/39)		
708	671	Potential structural protein	*Agrobacterium* phage Atu_ph07 (38.03/90)		

## Data Availability

The genome sequence of AP-J-162 was deposited in the GenBank with the nucleotide accession number.

## Supplementary Materials

The following supporting information can be downloaded at https://www.mdpi.com/article/10.3390/ijms25137388/s1.
