# Supplementary material for: Soil Giant Phage: Genome and Biological Characteristics of Sinorhizobium Jumbo Phage
Source: Int J Mol Sci. 2024 Jul 5;25(13):7388. doi: 10.3390/ijms25137388 (PMC11242549; doi:10.3390/ijms25137388)
Supplement: Supplementary file 1 [file ijms-25-07388-s001.zip › Figures S.pdf]

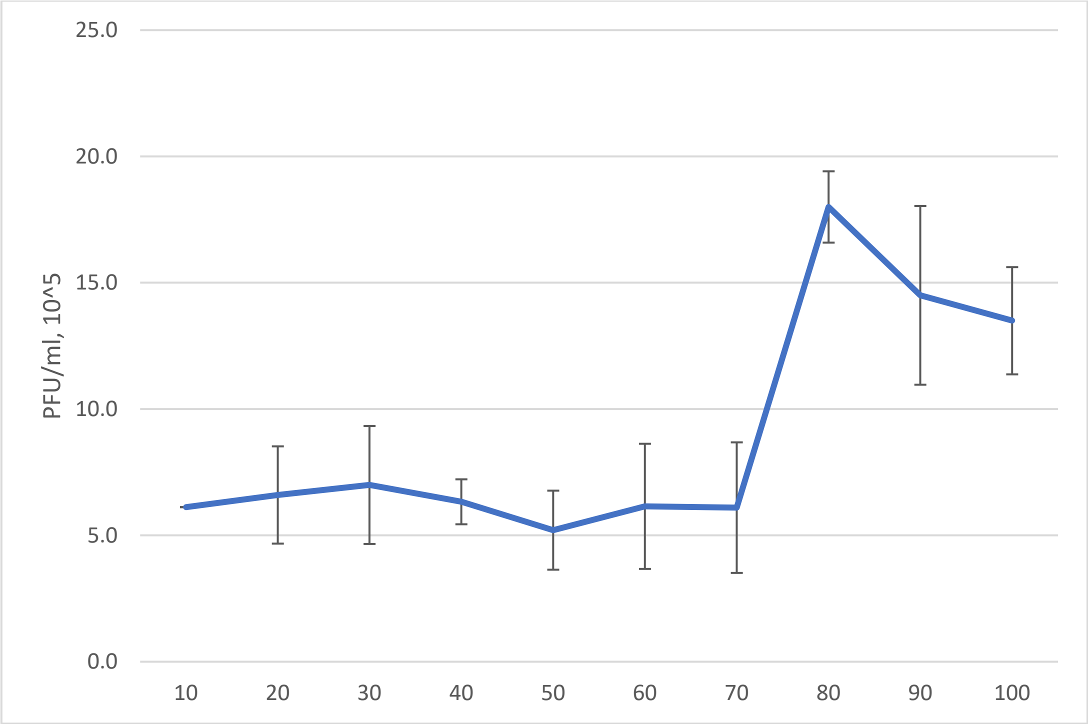

Figure S1. One-step growth curve of phage AP-J-162

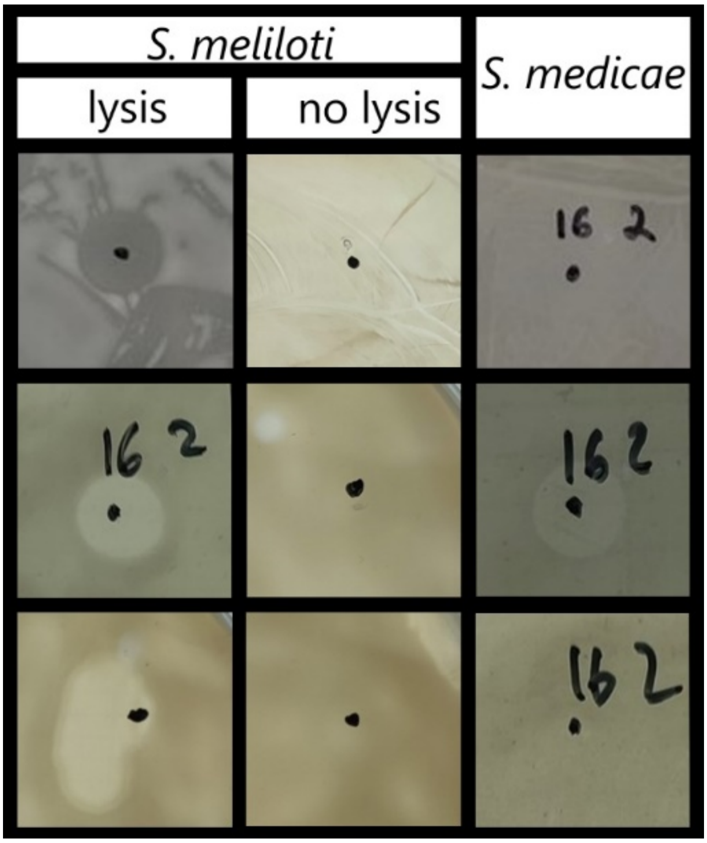

Figure S2. AP-J-162 lytic activity on *S. meliloti* and *S. medicae* strains obtained by spot test.

162 – phage working name of the AP-J-162; the black dots are the sites of phage application.
