# Supplementary material for: Soil Giant Phage: Genome and Biological Characteristics of Sinorhizobium Jumbo Phage
Source: Int J Mol Sci. 2024 Jul 5;25(13):7388. doi: 10.3390/ijms25137388 (PMC11242549; doi:10.3390/ijms25137388)
Supplement: Supplementary file 1 [file ijms-25-07388-s001.zip › Table S-1.Growth parameters of the S. meliloti Md3-4 culture infected with AP-J-162 and incubated in LB liquid medium.pdf]

Table S-1. Growth parameters of the S. meliloti Md3/4 culture infected with AP-J-162 and incubated in LB liquid medium.

**Growth parameters***			**Control (uninfected) Md3/4 culture**	**Infected Md3/4 culture**	
				**MOI 0.0003**	**MOI 0.001**
cell lysis			-	6	6
**growth phases**	lag	start	1.5	1.5	1.5
		duration	2	2	2
	log	start	3.5	3.5	3.5
		duration	16	9.5	9.5
	stationary	start	20	14	14
		duration	1	4	8
		OD600	0.9364	0.697	0.7148
secondary growth start time			-	22	20

* - in hours except OD600
