# Supplementary material for: Soil Giant Phage: Genome and Biological Characteristics of Sinorhizobium Jumbo Phage
Source: Int J Mol Sci. 2024 Jul 5;25(13):7388. doi: 10.3390/ijms25137388 (PMC11242549; doi:10.3390/ijms25137388)
Supplement: Supplementary file 1 [file ijms-25-07388-s001.zip › Table S-2. Codon usage and tRNAs.pdf]

Table S-2. Codon usage and tRNAs

Aminoacid/Codon in DNA (mRNA)		Frequency		Anticodon		Number of tRNA	
		**AP-J-162**	***S. meliloti***	**AP-J-162**	***S. meliloti***	**AP-J-162**	***S. meliloti***
Ala	GCG (GCG)	0.19	0.37	-	CGC		1
	GCA (GCA)	0.32	0.10	UGC	UGC	1	3
	GCT (GCU)	0.31	0.08	-	-		
	GCC (GCC)	0.18	0.45	GGC	GGC	1	1
Cys	TGT (UGU)	0.38	0.11	-	-		
	TGC (UGC)	0.63	0.89	GCA	GCA	2	1
Asp	GAT (GAU)	0.62	0.32	-	-		
	GAC (GAC)	0.38	0.68	GUC	GUC	2	2
Glu	GAG (GAG)	0.33	0.55	CUC	CUC	1	1
	GAA (GAA)	0.67	0.45	UUC	UUC	2	3
Phe	TTT (UUU)	0.44	0.11	AAA**	-	1	
	TTC (UUC)	0.56	0.89	GAA	GAA	3	1
Gly	GGG (GGG)	0.05	0.09	-	CCC		1
	GGA (GGA)	0.14	0.06	UCC	UCC	1	1
	GGT (GGU)	0.49	0.15	-	-		
	GGC (GGC)	0.32	0.69	GCC	GCC	2	1
His	CAT (CAU)	0.53	0.44	-	-		
	CAC (CAC)	0.47	0.56	GUG	GUG	2	1
Ile	ATA (AUA)	0.05	0.02	UAU**	-	1	
	ATT (AUU)	0.43	0.12	-	-		
	ATC (AUC)	0.53	0.86	GAU	GAU	3	3
Lys	AAG (AAG)	0.70	0.89	CUU	CUU	2	1
	AAA (AAA)	0.30	0.11	UUU	UUU	2	1
Leu	TTG (UUG)	0.23	0.05	CAA	CAA	2	1
	TTA (UUA)	0.02	0.00	UAA	UAA	1	1
	CTG (CUG)	0.16	0.39	CAG	CAG	1	1
	CTA (CUA)	0.10	0.01	UAG	UAG	1	1
	CTT (CUU)	0.27	0.12	-	-		
	CTC (CUC)	0.22	0.43	GAG	GAG	1	1
Met/fMet/Ile	ATG (AUG)	1	1	CAU	CAU	2	6
Asn	AAT (AAU)	0.44	0.28	-	-		
	AAC (AAC)	0.56	0.72	GUU	GUU	4	1
Pro	CCG (CCG)	0.20	0.69	CGG	CGG	1	1
	CCA (CCA)	0.43	0.02	UGG	UGG	2	1
	CCT (CCU)	0.21	0.06	-	-		
	CCC (CCC)	0.15	0.23	GGG	GGG	1	1
Gln	CAG (CAG)	0.54	0.94	CUG	CUG	1	1
	CAA (CAA)	0.46	0.06	UUG	UUG	2	1
Arg	AGG (AGG)	0.03	0.04	CCU	CCU	1	1
	AGA (AGA)	0.06	0.01	UCU	UCU	2	1
	CGG (CGG)	0.08	0.17	-	CCG		1
	CGA (CGA)	0.11	0.03	UCG**	-	1	

	CGT (CGU)	0.42	0.14	ACG	ACG	2	1
	CGC (CGC)	0.29	0.61	-	-		
Ser	AGT (AGU)	0.18	0.02	ACU**	-	1	
	AGC (AGC)	0.21	0.19	GCU	GCU	1	1
	TCG (UCG)	0.15	0.43	-	CGA		1
	TCA (UCA)	0.11	0.01	UGA	UGA	1	1
	TCT (UCU)	0.14	0.03	-	-		
	TCC (UCC)	0.21	0.32	-	GGA		1
Thr	ACG (ACG)	0.17	0.45	CGU	CGU	2	1
	ACA (ACA)	0.30	0.04	UGU	UGU	3	1
	ACT (ACU)	0.29	0.03	CUC**	-	1	
	ACC (ACC)	0.24	0.48	-	GGU		1
Val	GTG (GUG)	0.18	0.28	-	-		
	GTA (GUA)	0.20	0.04	UAC	UAC	2	1
	GTT (GUU)	0.44	0.13	-	-		
	GTC (GUC)	0.18	0.55	GAC	GAC	1	1
Trp	TGG (UGG)	1	1	CCA	CCA	2	1
Tyr	TAT (UAU)	0.53	0.45	-	-		
	TAC (UAC)	0.47	0.55	-	GUA		1
End	TGA (UGA)	0.33	0.50	-	UCA		1
	TAG (UAG)	0.05	0.10	CUA**	-	1	
	TAA (UAA)	0.63	0.39	-	-		
In total				41	45	66	55

* - core genes of bacteria host *S. meliloti* 1021 [35.36]; ** - unique phage AP-J-162 tRNAs
