# Supplementary material for: Soil Giant Phage: Genome and Biological Characteristics of Sinorhizobium Jumbo Phage
Source: Int J Mol Sci. 2024 Jul 5;25(13):7388. doi: 10.3390/ijms25137388 (PMC11242549; doi:10.3390/ijms25137388)
Supplement: Supplementary file 1 [file ijms-25-07388-s001.zip › Table S-3. ORFs of phage AP-J-162 belonging to different COG categories.pdf]

Table S-3. ORFs of phage AP-J-162 belonging to different COG categories

COG categories	Function	ORFs number in AP-J-162	Proteins encoded by ORFs of AP-J-162
C	energy production and conversion	3	Fe-S oxidoreductase (gp068), pyridine nucleotide-disulphide oxidoreductase (gp540), mega protein (inorganic pyrophosphatase gp600)
D	cell cycle control, cell division, chromosome partitioning	2	AAA family ATPase (gp103, 113)*
E	amino acid transport and metabolism	1	ornithine/lysine decarboxylase (gp280)
F	nucleotide acid transport and metabolism	9	thymidylate synthase (gp151), 5'(3')-deoxyribonucleotidase (gp162), pyrophosphatase (gp212), DNA/RNA non-specific endonuclease (gp432), thymidine kinase (gp516), ribonucleoside-diphosphate reductase (alpha – gp653, beta – gp654), ADP-ribose pyrophosphatase (gp691), nucleoside 2-deoxyribosyltransferase (gp695)
G	carbohydrate transport and metabolism	3	phosphoglycerate mutase (gp036), chitobiase/beta-hexosaminidase (gp079), endolysin (gp680)*
H	coenzyme transport and metabolism	12	NrdG organic radical activating enzyme (gp118), GTP cyclohydrolase I (gp121), dihydrofolate reductase (gp150), hydrolase (gp195), NADAR family protein (gp251)*, NAD kinase (gp269), 6-pyruvoyl tetrahydropterin synthase protein (gp558), kinase activity (gp667), ammonia-dependent NAD(+) synthetase (gp684), nicotinate phosphoribosyltransferase (gp686), nicotinamide mononucleotide adenylyltransferase (gp692), isochorismatase (gp693)*
J	translation, ribosomal structure and biogenesis	13	peptidyl-tRNA hydrolase (gp084, 295), cysteine-tRNA ligase (gp094), RNA ligase (gp097, 361), N-acetyltransferase (gp099), N-formylmethionyl-tRNA deformylase (gp154), carbon-nitrogen ligase activity, with glutamine as amido-N-donor (gp239), 30S ribosomal protein S21 (gp532), translation initiation factor IF-3 (gp547), 50S ribosomal protein L7/L12 (gp556), tRNA nucleotidyltransferase (gp566), aminoacyl-tRNA hydrolase activity (gp671)
K	transcription	2	regulation of transcription, DNA-dependent (gp061), (p)ppGpp synthase/hydrolase (gp505)*
L	replication, recombination and repair	23	DNA double-strand break repair ATPase (gp010), exonuclease activity (gp035), recombination-related endonuclease (gp041,

			044), exonuclease (gp054, 599), hydrolase activity (gp108), catalytic activity (gp124), DNA polymerase (gp112, 127, 206), UV damage endonuclease UvsE (gp131), DNA ligase (gp135), replication factor C (gp197), DEAD/DEAH box helicase (gp288), DNA topoisomerase (gp551, 553), ATP-dependent DNA helicase uvsW (gp638), recombination, repair and ssDNA binding protein UvsY (gp643), RecA RecA/RadA recombinase (gp645), thermostable 8-oxoguanine DNA glycosylase (gp021)*, DNA primase/helicase (gp619)*, DNA primase(gp620)*
M	cell wall/membrane/envelope biogenesis	8	transferase activity (gp048, 122), AAA family ATPase (gp103, 113)*, prohead core scaffolding protein and protease (gp024)* glycosyltransferase (gp228), UDP-2,3-diacylglucosamine diphosphatase (gp229), L-alanyl-D-glutamate peptidase (gp289),
N	cell motility	1	type II secretion system F family protein (gp319)*
O	posttranslational modification, protein turnover, chaperones	21	prohead core scaffolding protein and protease (gp024)*, [formate-C-acetyltransferase]-activating enzyme activity (gp062), glutaredoxin (gp106, 652), Clp proteins (gp153, 175, 271, 515, 548, 661), DnaJ protein (gp155), NUDIX domain-containing protein (gp174), co-chaperone GroES (gp204), extracellular serine proteinase precursor (gp224), lysozyme-like protein (gp243), ATP-grasp domain-containing protein (gp252), methyltransferase domain (gp310), CRISPR/Cas system-associated protein Cas4 (gp489), ADP-ribosylglycohydrolase (gp499), Hsp20 family protein (gp517), thioredoxin (gp642)
P	inorganic ion transport and metabolism	1	ryanodine receptor Ryr domain (gp137)
Q	secondary metabolites biosyntesis, transport and catabolism;	1	5-bromo-4-chloroindolyl phosphate hydrolysis family protein (gp571)*
R	general function prediction only	8	radical SAM protein (gp069), glycoside hydrolase (gp270), metallophosphatase (gp293), concanavalin A-like lectin/glucanases superfamily (gp355), 5-bromo-4-chloroindolyl phosphate hydrolysis family protein (gp571)*ASCH domain-containing protein (gp635), autotransporter (gp650), isochorismatase (gp693)*
S	function unknown	1	putative internalin (gp567)
T	signal transduction mechanisms	7	protein serine-threonine phosphatase (gp081), AAA family ATPase (gp103, 113)*, PhoH-like protein (gp192), putative anti-sigma factor (gp276), (p)ppGpp synthase/hydrolase

			(gp505)*, multi-sensor signal transduction histidine (gp608),
U	intracellular trafficing, secretion and vesicular transport	2	type II secretion system F family protein (gp319)*, PAAR domain-containing protein (gp582),
V	defense mechanisms	7	thermostable 8-oxoguanine DNA glycosylase (gp021)*, HNH endonuclease (gp037, 080, 543), NADAR family protein (gp251)*, NUDIX domain-containing protein (gp534), toxic anion resistance protein (gp570),
W	extracellular structures	1	type II secretion system F family protein (gp319)*
X	mobilome: prophages, transposons	7	terminase (gp002, 005, 648), holin (gp138), DNA primase/helicase (gp619)*, DNA primase(gp620)*, endolysin (gp680)*,

* - proteins belonged to more than one COG categories
