# Supplementary material for: Soil Giant Phage: Genome and Biological Characteristics of Sinorhizobium Jumbo Phage
Source: Int J Mol Sci. 2024 Jul 5;25(13):7388. doi: 10.3390/ijms25137388 (PMC11242549; doi:10.3390/ijms25137388)
Supplement: Supplementary file 1 [file ijms-25-07388-s001.zip › Table S-4. Characteristics of the phages that were used for phylogenetic analyses.pdf]

Table S-4. Bacteriophages involved in phylogenetic analyses

Phage	Classification	Type	Genome length, k.b.p.	RefSeq
Sinorhizobium phage phiN3	Emdodecavirus	myovirus	206.7	NC_028945.1
Sinorhizobium phage phiM12	Emdodecavirus	myovirus	194.7	NC_027204.1
Sinorhizobium phage phiM7	Emdodecavirus	myovirus	188.4	NC_041929.1
Agrobacterium phage Atu_ph07	Polybotosvirus	myovirus	490.4	NC_042013.1
Bacillus phage G	Donellivirus	myovirus	497.5	NC_023719.1
Caulobacter phage CcrColossus	Dolichocephalovirinae; Colossusvirus	siphovirus	280.0	NC_019406.1
Cronobacter phage vB_CsaM_GAP32	Mimasvirus	myovirus	358.7	NC_019401.1
Enterobacteria phage T4	Straboviridae; Tevenvirinae; Tequatrovirus	myovirus	168.9	NC_000866.4
Enterobacteria phage vB_KleM-RaK2	Alcyoneusvirus	myovirus	345.8	NC_019526.1
Escherichia phage 121Q	Asteriusvirus	myovirus	348.5	NC_025447.1
Escherichia phage PBECO 4	Asteriusvirus	myovirus	348.1	NC_027364.1
Klebsiella phage K64-1	Alcyoneusvirus	myovirus	346.6	NC_027399.1
Prochlorococcus phage P-SSM2	Kyanoviridae; Salacisavirus	myovirus	252.4	NC_006883.2
Pseudomonas phage Lu11	unclassified	myovirus	280.5	NC_017972.1
Pseudomonas phage OBP	Petsuvirus	myovirus	284.8	NC_016571.1
Pseudomonas phage PA7	Phikzvirus	myovirus	266.7	NC_042060.1
Pseudomonas phage Phabio	Phabiovirus	-	309.2	NC_062582.1
Ralstonia phage RSL1	Mieseafarmvirus	myovirus	231.3	NC_010811.2
Salicola phage SCTP-2	unclassified	myovirus	440.0	MF360958.1
Serratia phage BF	Eneladusvirus	myovirus	357.2	NC_041917.1
Sphingomonas phage PAU	unclassified	myovirus	219.4	NC_019521.1
Synechococcus phage Bellamy	Kyanoviridae; Bellamyvirus	myovirus	204.9	NC_047838.1
Vibrio phage KVP40	Straboviridae; Schizotequatrovirus	myovirus	244.8	NC_005083.2
Xanthomonas phage XacN1 DNA	unclassified	myovirus	384.7	AP018399.1
Yersinia phage fHe-Yen9-04	Eneladusvirus	myovirus	354.4	NC_042116.1
