# Supplementary figures and images for: Soil Giant Phage: Genome and Biological Characteristics of Sinorhizobium Jumbo Phage
Source: Int J Mol Sci. 2024 Jul 5;25(13):7388. doi: 10.3390/ijms25137388 (PMC11242549; doi:10.3390/ijms25137388)

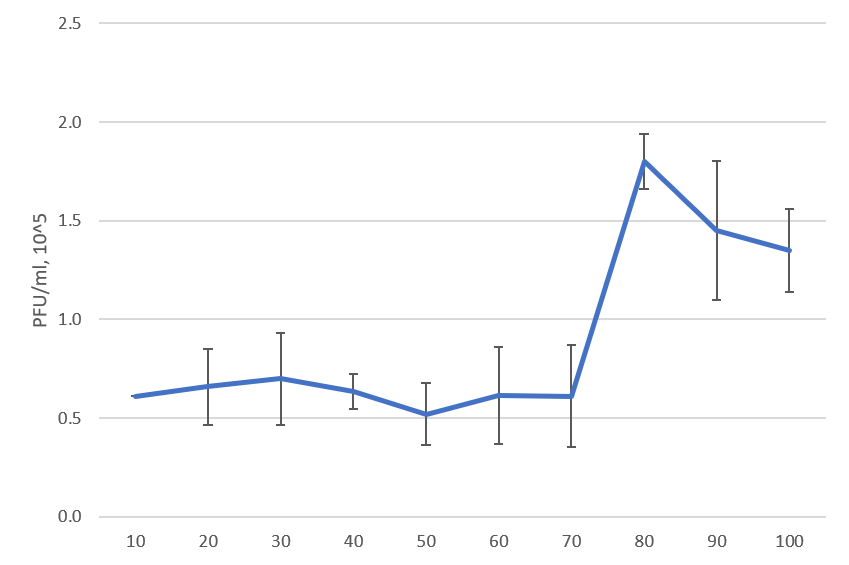

Supplement: Supplementary file 1 [file ijms-25-07388-s001.zip › Figure S-1.png]

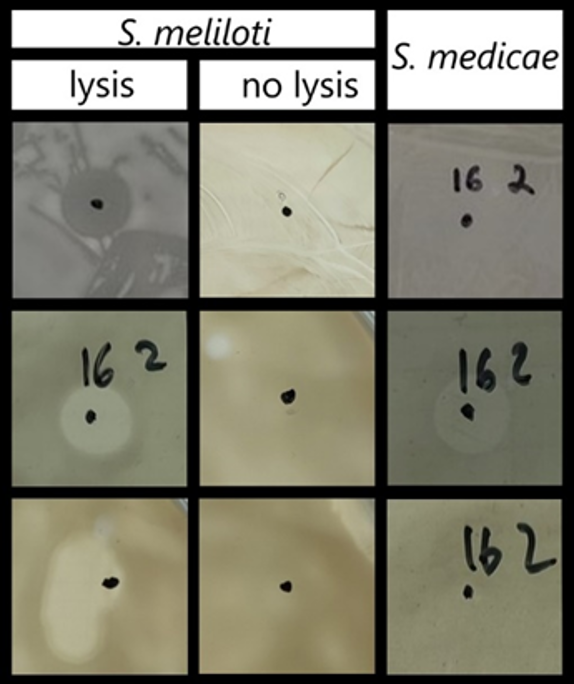

Supplement: Supplementary file 1 [file ijms-25-07388-s001.zip › Figure S-2.png]
